# Atmospheric Turbulence Phase Reconstruction via Deep Learning Wavefront Sensing

**DOI:** 10.3390/s24144604

**Published:** 2024-07-16

**Authors:** Yutao Liu, Mingwei Zheng, Xingqi Wang

**Affiliations:** 1 School of Information Science and Engineering, Yanshan University, Qinhuangdao 066004, China; zhengmw960218@163.com (M.Z.); wangxq@stumail.ysu.edu.cn (X.W.); 2 The Key Laboratory for Special Fiber and Fiber Sensor of Hebei Province, Qinhuangdao 066004, China

**Keywords:** atmospheric turbulence, phase reconstruction, deep learning, U-Net network

## Abstract

The fast and accurate reconstruction of the turbulence phase is crucial for compensating atmospheric disturbances in free-space coherent optical communication. Traditional methods suffer from slow convergence and inadequate phase reconstruction accuracy. This paper introduces a deep learning-based approach for atmospheric turbulence phase reconstruction, utilizing light intensity images affected by turbulence as the basis for feature extraction. The method employs extensive light intensity-phase samples across varying turbulence intensities for training, enabling phase reconstruction from light intensity images. The trained U-Net model reconstructs phases for strong, medium, and weak turbulence with an average processing time of 0.14 s. Simulation outcomes indicate an average loss function value of 0.00027 post-convergence, with a mean squared error of 0.0003 for individual turbulence reconstructions. Experimental validation yields a mean square error of 0.0007 for single turbulence reconstruction. The proposed method demonstrates rapid convergence, robust performance, and strong generalization, offering a novel solution for atmospheric disturbance correction in free-space coherent optical communication.

## 1. Introduction

Free-space coherent optical communication is recognized as a critical technology to surmount current high-speed communication limitations due to its superior data rates and information capacity [1,2,3]. During the transmission of a signal beam through free space, atmospheric turbulence-induced refractive index fluctuations lead to amplitude and phase distortions, substantially impairing the communication quality of free-space coherent optical communication systems [4,5]. This phenomenon is particularly pronounced in long-distance space-to-ground communications, where the signal beam undergoes long-distance atmospheric channel propagation, leading to an incomplete phase, distorted wavefronts, and thus a reduced communication bit error rate during signal demodulation at the receiving end. Consequently, the impact of atmospheric turbulence on space-to-ground communication is particularly severe [6]. The principle behind the effect of atmospheric turbulence on the signal beam is the addition of a random turbulence phase to the signal beam. Therefore, the accurate reconstruction of the turbulence phase is crucial for addressing atmospheric disturbance challenges in the field of free-space coherent optical communication.

Adaptive optic methods are a common and effective approach to compensate for spatial phase distortions. Mature adaptive optic technologies are categorized into two main types based on the utilization of wavefront sensing: conventional adaptive optics and wavefront sensor-less adaptive optics [7,8]. The fundamental approach of the conventional adaptive optic method entails the use of wavefront sensing instruments, such as Shack–Hartmann wavefront sensors or curvature wavefront sensors, to measure the wavefront aberrations in the signal beam. Subsequently, these aberrations are corrected by a wavefront compensation system composed of deformable mirrors. Nevertheless, scintillation effects caused by intense turbulence can disrupt the wavefront of the signal beam, thereby compromising the accuracy of wavefront sensor measurements [9,10].

The wavefront sensor-less adaptive optics approach cannot directly measure wavefront information. Consequently, optimization algorithms such as GA, PSO, RUN, or SPGD are employed to iteratively optimize the control signal of the wavefront corrector, using metrics such as image clarity, Strehl ratio, or coupling efficiency as the objective function to achieve optimal phase compensation [11,12,13,14]. Nonetheless, the iterative process of these optimization algorithms constrains the control bandwidth of wavefront sensor-less adaptive optic systems. The phase inversion method directly reconstructs the distorted wavefront from the far-field image. However, conventional phase inversion methods based on the Gerchberg–Saxton algorithm [15] or the phase difference method [16] necessitate multiple iterations to resolve wavefront phase information, which fails to satisfy the real-time demands of spatial phase distortion compensation.

Due to its outstanding advantage in extracting deep feature information from images, deep neural networks are expected to achieve the direct calculation of the phase from far-field images to the wavefront, improving the real time and accuracy of phase inversion [17,18,19,20]. In this study, a method is presented for reconstructing the turbulence phase based on the U-Net network. The method uses intensity images affected by turbulence as feature extraction objects and utilizes a large dataset of intensity-phase samples under different turbulence intensities for training, achieving turbulence phase reconstruction based on intensity images. To better deal with complex and diverse atmospheric conditions, the turbulence phase was extracted from Gaussian beams affected by different turbulence intensities (*C_n_*^2^ = 5 × 10^−17^, *C_n_*^2^ = 5 × 10^−15^, *C_n_*^2^ = 1 × 10^−13^). Moreover, this paper further verified the effectiveness of the proposed method by building an experimental system. The results demonstrate that the U-Net-based approach offers a rapid and accurate method for reconstructing atmospheric turbulence phases, presenting a novel strategy for wavefront phase compensation. The proposed method exhibits strong generalization ability and robustness, enabling accurate and rapid wavefront phase reconstruction across diverse turbulence intensities, which is advantageous for the deployment of free-space coherent optical communication systems.

## 2. Atmospheric Turbulence Model

To evaluate the detrimental impact of atmospheric turbulence on coherent optical communication systems, a quantitative analysis of the stochastic distribution properties of both the amplitude and phase after the transmission of the signal beam through atmospheric turbulent channels is essential. Light’s propagation through the atmosphere exhibits a refractive index power spectral density as a stochastic process. The creation of an atmospheric turbulence phase screen utilizes the low-frequency compensation power spectrum inversion method to create an independent stochastic process that mirrors the refractive index power spectral density of atmospheric turbulence, facilitating numerical simulations of the spatial phase distortion caused by atmospheric turbulence [21,22]. In addressing this limitation, third-order harmonic compensation was introduced across varying turbulence intensities. The process of generating phase screens using the power spectral method can be expressed as follows:

(1) $$\varphi (j\Delta x,l\Delta y)=\sum_{m=0}^{N_{x}}\sum_{n=0}^{N_{y}}R(k_{x},k_{y})\sqrt{\Delta k_{x},\Delta k_{y}}\sqrt{2\pi k^{2}\Delta z\Phi_{n}(m\Delta k_{x},n\Delta k_{y})}\exp(2\pi i(\frac{jm}{N_{x}}+\frac{ln}{N_{y}})),$$

where Δ*x* and Δ*y* represent the sampling interval of the phase screen in the *x* and *y* directions, *R*(*k_x_*, *k_y_*) is a complex Gaussian random matrix, Φ*_n_*(*m*Δ*k_x_*,*n*Δ*k_y_*) is the power spectrum function, the wave number *k* is given by *k* = 2π/λ, *k_x_* = 2π/*N_x_* and *k_y_* = 2π/*N_y_* are wave number increments, and Δ*z* represents the distance between phase screens. However, the power spectrum inversion method suffers from an insufficient number of low-frequency components. To solve this problem, we added third-order harmonic compensation to different turbulence intensities. The subharmonics were generated as follows:

(2) $$\varphi_{SH}(j\Delta x,l\Delta y)=\sum_{p=1}^{N_{p}}\sum_{m=-1}^{1}\sum_{n=-1}^{1}R(k_{xp},k_{yp})\sqrt{\Delta k_{xp},\Delta k_{yp}}\sqrt{2\pi k^{2}\Delta z\Phi_{n}(\Delta k_{xp},\Delta k_{yp})}\exp(2\pi i(\frac{jm}{3^{p}N_{x}}+\frac{ln}{3^{p}N_{y}})),$$

where *P* is the series of subharmonics and the frequency intervals of the subharmonics are Δ*k_xp_* = Δ*k_x_*/3*^p^* and Δ*k_yp_* = Δ*k_y_*/3*^p^*, respectively. The phase screen after the subharmonic compensation is obtained by adding (1) and (2).

**Figure 1:**
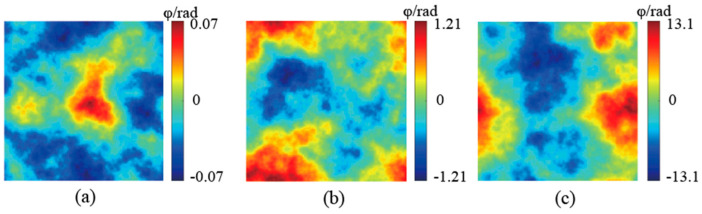
Simulation results of atmospheric turbulence phase screen under different turbulence intensities. (**a**) Weak turbulence (*C_n_*^2^ = 5 × 10^−17^ m^−2/3^); (**b**) moderate turbulence (*C_n_*^2^ = 5 × 10^−15^ m^−2/3^); (**c**) strong turbulence (*C_n_*^2^ = 1 × 10^−13^ m^−2/3^).

Based on the methodology outlined above, the impact of atmospheric turbulence on the beam can be likened to introducing random phase noise, with the extent of spatial phase distortion induced by atmospheric turbulence being quantified by the atmospheric refractive index structure constant *C_n_*^2^. The simulation results of atmospheric turbulence phase screens under varying *C_n_*^2^ values are illustrated in Figure 1. The simulation assumes an optical wavelength of 532 nm, with a grid point count of N = 128 and a phase screen width of 0.4 m. Figure 1a–c depict phase screens corresponding to weak (*C_n_*^2^ = 5 × 10^−17^ m^−2/3^), moderate (*C_n_*^2^ = 5 × 10^−15^ m^−2/3^), and strong (*C_n_*^2^ = 1 × 10^−13^ m^−2/3^) turbulence, respectively. As the *C_n_*^2^ value increases, the severity of spatial phase distortion induced by atmospheric turbulence escalates. While weak turbulence results in relatively mild phase distortion, strong turbulence can induce profound spatial phase distortion in the beam, significantly impairing the light’s wavefront.

To simulate the transmission process of Gaussian light in the atmosphere, this paper adopts the most important fundamental mode of Gaussian light in Gaussian light, and assumes that Gaussian light propagates in the Z direction. The optical field expression of the Gaussian beam can be expressed as follows [23]:

(3) $$U(x,y,z)=U_{0}\frac{\omega_{0}}{\omega (z)}\exp(-\frac{r^{2}}{\omega^{2}(z)})\times \exp\{-i[kz-\tan^{-1}\frac{z}{z_{R}}+\frac{kr^{2}}{2R(z)}]\},$$

where *U*_0_ is a constant; the cross-sectional radius *r*^2^ is defined as *r*^2^ = *x*^2^ + *y*^2^; *ω*(*z*) represents the cross-sectional radius at position *z*, where *ω*_0_ is the waist radius of the fundamental mode Gaussian beam; and *R*(*z*) is the wavefront curvature radius at point *z*.

Assuming that the spatial phase distortion induced by a random continuous atmosphere is ascribed to an infinitely thin atmospheric turbulence phase screen positioned at the midpoint of the propagation path, the transmission of Gaussian beams through atmospheric turbulence can be modeled as two sequential processes: propagation in vacuum and modulation by the phase screen. Equation (3) can be divided into two parts: the amplitude factor before the multiplication sign and the phase factor after the multiplication sign. The main impact of atmospheric turbulence on the Gaussian beam is on the phase. After propagating a certain distance, the accumulated phase fluctuations caused by turbulence will result in changes in the complex amplitude, leading to distortion in the light intensity. The optical field expression of the Gaussian beam affected by turbulence is given by

(4) $$U(x,y,z)=U_{0}\frac{\omega_{0}}{\omega (z)}\exp(-\frac{r^{2}}{\omega^{2}(z)})\times \exp\{-i[kz-\tan^{-1}\frac{z}{z_{R}}+\frac{kr^{2}}{2R(z)}]\}\times \exp[i\varphi (x,y)].$$

**Figure 2:**
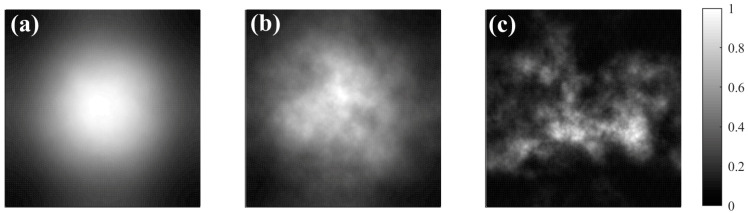
The intensity distributions simulated under different turbulence intensities. (**a**) Weak turbulence (*C_n_*^2^ = 5 × 10^−17^ m^−2/3^); (**b**) moderate turbulence (*C_n_*^2^ = 5 × 10^−15^ m^−2/3^); (**c**) strong turbulence (*C_n_*^2^ = 1 × 10^−13^ m^−2/3^).

In this study, the power spectrum inversion method was used to simulate the phase screens, with the following parameters: the wavelength of the light wave was *λ* = 532 nm, the initial radius of the Gaussian beam was *ω*_0_ = 20 mm, the number of grid points was N = 128, and the width of the phase screens was 0.4 m. Before the network training, the image was grayscaled. The intensity distributions simulated for different turbulence intensities are shown in Figure 2.

Figure 2 illustrates that the distortion of the light beam escalates with the augmentation of turbulence intensity. At a turbulence intensity of *C_n_*^2^ = 5 × 10^−17^ m^−2/3^, the light spot displayed measly drift. When the turbulence intensity was *C_n_*^2^ = 5 × 10^−15^ m^−2/3^, the light spot showed slight spreading without discernible distortion. However, at a turbulence intensity of *C_n_*^2^ = 1 × 10^−13^ m^−2/3^, the light spot center experienced severe drift, and the initially circular shape transformed into an irregular form. This transformation is attributed to the increase in irregular turbulent phase contributions to the light beam as the turbulence intensity grows, thereby enhancing phase and intensity fluctuations, which resulted in substantial beam distortion.

## 3. U-Net Model

**Figure 3:**
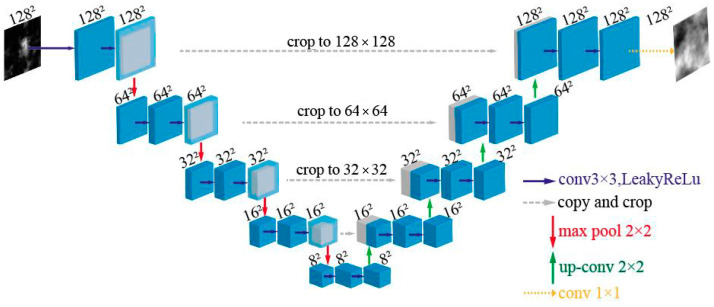
U-Net network structure.

The U-Net architecture employed in this study is illustrated in Figure 3. It consists of four downsampling stages and four upsampling stages. Each stage contains two convolutional layers. In contrast to the standard U-Net, this network utilizes padding to prevent a reduction in the image size during convolution. Consequently, the horizontal gray arrows directly concatenate two feature maps using the “cat” operation. During the downsampling process, which is represented by the red arrows, a 2 × 2 max pooling operation was applied to reduce the size of the feature maps. Conversely, during the upsampling process, which is represented by the green arrows, a 2 × 2 transpose-convolution operation was used.

The network input was a single-channel two-dimensional image representing a Gaussian beam affected by atmospheric turbulence. To reduce the computational complexity, the image size was initially reduced to 128 × 128 pixels. The downsampling process involved applying a convolution (conv1) with a 3 × 3 kernel, stride of 1, and padding of 1 to obtain 64 feature maps with a size of 128 × 128. These feature maps were then downsampled to 64 feature maps with a size of 64 × 64 using a 2 × 2 max pooling operation. This process was repeated with conv2, resulting in 128 feature maps of size 64 × 64 being downsampled to 128 feature maps of size 32 × 32. Subsequently, conv3 generated 256 feature maps of size 32 × 32, which were further downsampled to 256 feature maps of size 16 × 16. Conv4 produced 512 feature maps with a size of 16 × 16, which were downsampled to 512 feature maps with a size of 8 × 8. Finally, conv5 generated 1024 feature maps of size 8 × 8, indicating the completion of the downsampling process. The network then proceeded with an upsampling process to restore the original image size.

During the first upsampling stage, the feature maps from conv4 were upsampled to 512 feature maps with dimensions of 16 × 16. These upsampled feature maps were concatenated (cat) with the corresponding feature maps from conv4, forming 1024 feature maps of size 16 × 16. The resulting feature maps passed through conv6 to generate 512 feature maps with a size of 16 × 16. A similar process occurred in the subsequent upsampling stages. The feature maps from conv3 were upsampled to 256 feature maps of size 32 × 32 and concatenated with the corresponding feature maps from conv3 to form 512 feature maps of size 32 × 32 after passing through conv7. The feature maps from conv2 were then upsampled to 128 feature maps of size 64 × 64 and concatenated with the corresponding feature maps from conv2 to form 256 feature maps of size 64 × 64 after passing through conv8. Finally, the feature maps from conv1 were upsampled to 64 feature maps of size 128 × 128 and concatenated with the corresponding feature maps from conv1 to form 128 feature maps of size 128 × 128 after passing through conv9. The prediction layer outputs the turbulence phase, which affects Gaussian beam transmission. The entire process of the U-Net network allows the accurate prediction of the turbulence phase from the intensity images affected by atmospheric turbulence.

The U-Net network uses a loss function composed of the predicted and actual turbulent phases to evaluate its performance. The sample error loss function used in this study was

(5) $$Loss=-\frac{1}{N}\sum_{i=1}^{N}\left(y_{i}\log({\hat{y}}_{i})+(1-y_{i})\log(1-{\hat{y}}_{i})\right)$$

where *N* represents the number of samples, *y_i_* represents the actual turbulent phase, and *ŷ_i_* represents the turbulent phase predicted by the U-Net model. First, for each sample, we calculated the loss of the true label and the predicted result. Then, we summed the losses of all the samples, and finally, we divided the sum by the number of samples to obtain the average loss. The MSE was used to measure the quality of the model predictions:

(6) $$MSE=\frac{1}{m}\sum_{i=1}^{m}{\left(y_{i}-{\hat{y}}_{i}\right)}^{2}$$

where *m* represents the total number of pixels in the predicted and actual turbulence phase images.

## 4. Phase Reconstruction of Atmospheric Turbulence

To verify the effectiveness of the method, we used the atmospheric turbulence phase screen to modulate the Gaussian beam on the transmission path and then used the U-Net network to reconstruct the turbulent phase in the distorted Gaussian beam. The turbulent phase was then reconstructed. To ensure that the model had a good generalization ability, 6000 training sets and 2400 test sets were constructed for the three turbulence intensities of *C_n_*^2^ = 5 × 10^−17^ m^−2/3^, *C_n_*^2^ = 5 × 10^−15^ m^−2/3^, and *C_n_*^2^ = 1 × 10^−13^ m^−2/3^. The training sets were used to train the U-Net model, and the test set was used to test the trained model. The method was considered to be effective if the turbulence phase output by the U-Net model was consistent with the actual turbulence phase after a distorted Gaussian beam affected by atmospheric turbulence was input into the U-Net model.

The training process encompasses two primary stages: forward propagation and backward propagation. During forward propagation, the training parameters of the U-Net network are adjusted, whereas backward propagation focuses on optimizing the network parameters based on the loss function. The U-Net network was trained using Python on a system equipped with an NVIDIA GTX 4080 GPU, employing an Adam optimizer with an initial learning rate of 10^−3^, a batch size of 50, and a total of 2500 training epochs. After a certain number of rounds of training, the network loss function reaches a fitting point. At this point, the network model training ends, and the weights are saved for the atmospheric turbulence phase reconstruction task.

**Figure 4:**
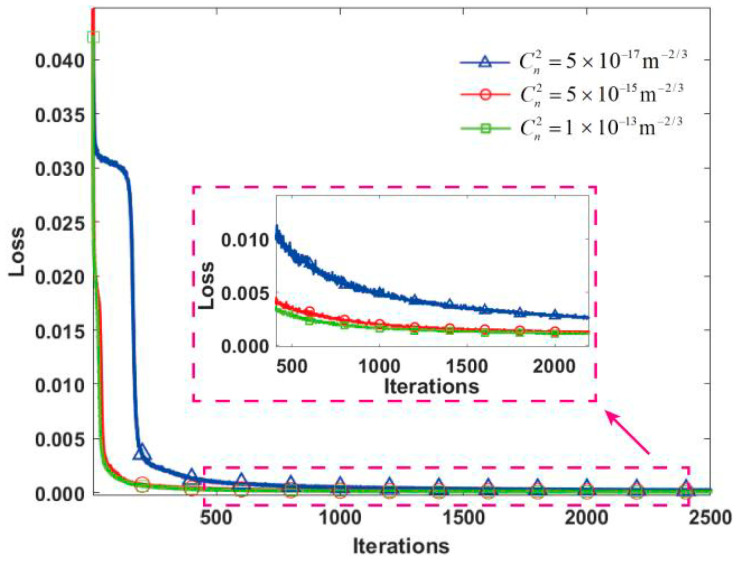
Loss function curves for different turbulence intensities. The picture in the red dotted box pointed to by the arrow is the enlarged picture of the curve after the 500th iteration.

Figure 4 presents the convergence curves of three different turbulence intensity loss functions. As shown, the U-Net model had almost the same reconstruction performance for different turbulence intensities, indicating that the model has a strong generalization ability. Furthermore, it can be observed that there are burrs in the enlarged curve image. The reason is that the loss function value during the training process is calculated based on the comparison between the reconstructed turbulence phase and the actual turbulence phase in each iteration. The turbulence phase reconstructed with each iteration of the network cannot be guaranteed to be closer to the actual turbulence phase than the last time. Therefore, the value of the loss function is constantly fluctuating locally, but it is still monotonically decreasing overall.

**Figure 5:**
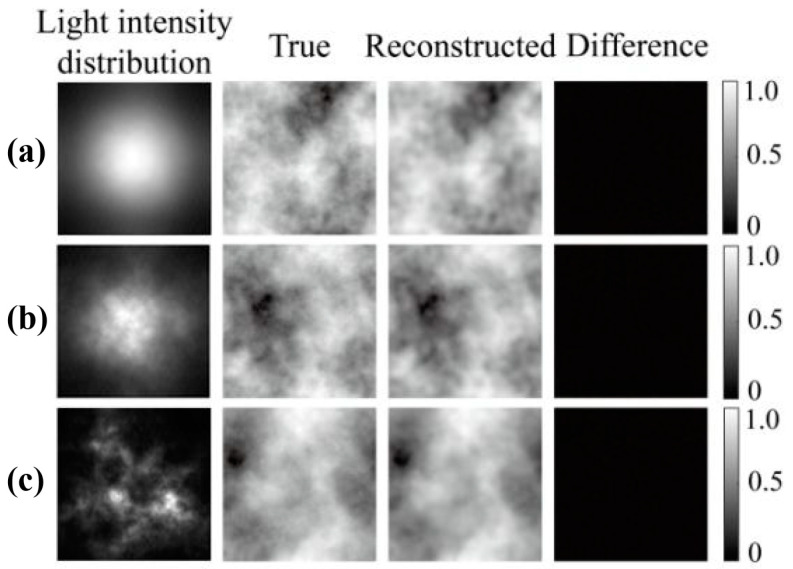
Actual and reconstructed turbulent phase screens and the difference between them under various turbulence intensities. (**a**) *C_n_*^2^ = 5 × 10^−17^ m^−2/3^, and the MSE = 0.00033; (**b**) *C_n_*^2^ = 5 × 10^−15^ m^−2/3^, and the MSE = 0.00032; (**c**) *C_n_*^2^ = 1 × 10^−13^ m^−2/3^, and the MSE was 0.00029.

The test results of the model for the three turbulence intensities are shown in Figure 5. The first, second, and third rows present the simulation results for turbulence intensities of *C_n_*^2^ = 5 × 10^−17^ m^−2/3^, *C_n_*^2^ = 5 × 10^−15^ m^−2/3^, and *C_n_*^2^ = 1 × 10^−13^ m^−2/3^, respectively. The last image in each row shows the reconstructed turbulence. The difference between the reconstructed image and the actual image is presented in Figure 5. Under the three turbulence intensities, the MSEs from the first row to the third row are 0.00033, 0.00032, and 0.00029, respectively. According to the network reconstruction results, the difference between the reconstructed and actual phase screens was very small. The proposed model had the best reconstruction effect for strong turbulence, presumably because the images in the case of strong turbulence had more obvious features for the network to identify and extract.

**Figure 6:**
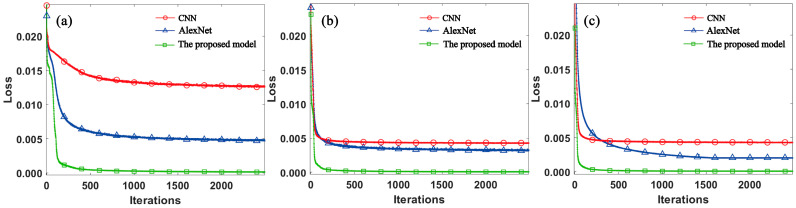
Loss function value decline curves under different turbulence intensities of CNN, the AlexNet, and the proposed model. (**a**) Weak turbulence (*C_n_*^2^ = 5 × 10^−17^ m^−2/3^); (**b**) moderate turbulence (*C_n_*^2^ = 5 × 10^−15^ m^−2/3^); (**c**) strong turbulence (*C_n_*^2^ = 1 × 10^−13^ m^−2/3^).

To demonstrate the advantages of the U-Net-based method in turbulent phase reconstruction, we compared it with the CNN [15] and AlexNet [16]. The decline curves of the loss function of the three models after training under different turbulence intensities are shown in Figure 6. All three networks were trained using the same dataset, and the number of iterations was set to 2500. As shown in Figure 6a–c, we display the loss function descent curves for the three network models under *C_n_*^2^ = 5 × 10^−17^ m^−2/3^, *C_n_*^2^ = 5 × 10^−15^ m^−2/3^, and *C_n_*^2^ = 1 × 10^−13^ m^−2/3^ turbulence intensities, respectively. The red, blue, and green color curves represent the loss function values of CNN, the AlexNet, and the proposed model decreasing as the number of iterations increases, respectively. Lower loss values indicate that the network is more accurate in phase reconstruction.

The CNN and the AlexNet had slower convergence than the proposed model; even after 2500 iterations, their loss function values exhibited a downward trend, indicating that these two networks require more iterations to reach convergence. In contrast, the proposed model had already reached convergence after 2500 iterations, and its loss function value was lower than those of the other two networks. These results indicate that the proposed model achieves excellent turbulence phase reconstruction with significantly fewer iterations.

In addition, the time required to predict the turbulent phase is one of the criteria for evaluating the ability to extract turbulent information. Therefore, we recorded the average amounts of time required by the three models to predict the turbulent phase. As shown in Table 1, the CNN and AlexNet models both required more time; they took approximately 0.31 and 0.29 s, respectively. In comparison, the proposed model took approximately 0.14 s to reconstruct the turbulent phase. In summary, compared with the other two models, the loss function value of the model proposed in this paper has dropped by more than 20 times, and the phase reconstruction time has been reduced by more than half, which is significantly faster than the turbulence change rate using Greenwood frequency as the evaluation index. This indicates that the reconstruction time is meeting the requirement for fast reconstruction.

## 5. Experiment Results

**Figure 7:**
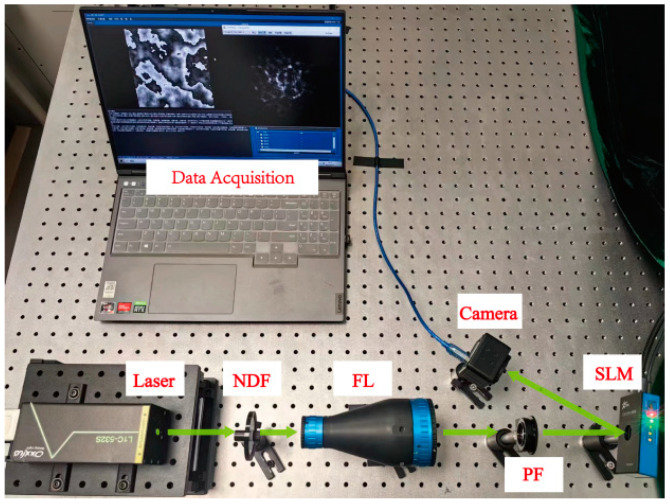
Experimental system structure. NDF, Neutral Density Filter; FL, Field Lens; PF, Polarizing Filter; SLM, Spatial Light Modulator.

To evaluate the effectiveness of the proposed method for turbulence phase reconstruction, an experimental platform was established to gather empirical data. Illustrated in Figure 7, the light emitted from the laser source (532 nm) passes through a series of optical components, including a neutral density filter (NDF) and a field lens (FL), resulting in optical power attenuation and beam expansion. Subsequently, the light beam traverses a polarizing filter (PF) before impinging upon the spatial light modulator (SLM). The SLM is programmed with an atmospheric turbulence phase screen of varying turbulence intensity to induce wavefront aberrations [26]. Ultimately, the distorted beam, manipulated by the SLM, is captured by the camera. The images captured by the camera are acquired using dedicated data acquisition software on the computer, depicted in the interface in Figure 7. On the left-hand side of the computer screen, the atmospheric turbulence phase screen loaded onto the SLM is displayed, while the corresponding distorted light intensity image is presented on the right-hand side. These images, collected by the computer, constitute the dataset utilized for training and validating the proposed model.

**Figure 8:**
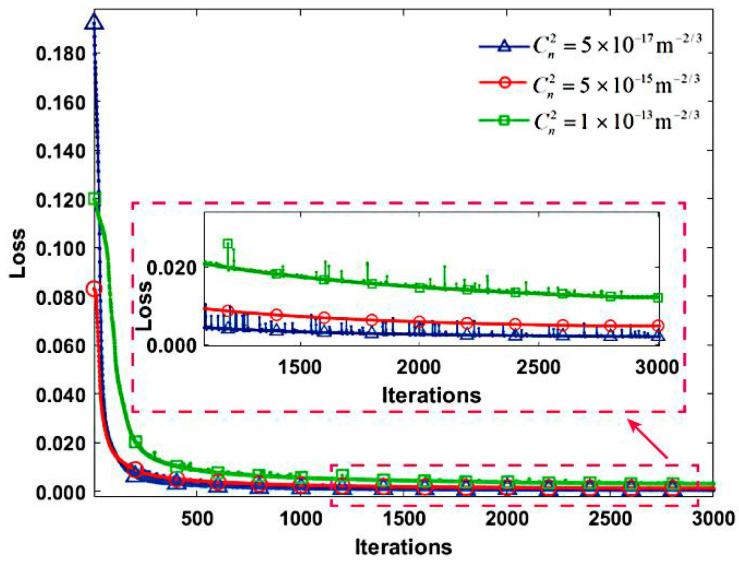
The loss function curves under three different turbulence intensities. The blue, red, and green curves correspond to the loss function curves under weak, moderate, and strong turbulence intensities, respectively. The red box indicated by the red arrow shows the amplified loss function curves after 1200 iterations.

This paper utilizes a dataset comprising 2000 images for training across three distinct turbulence intensities, supplemented by an additional 300 images allocated for testing purposes. Throughout the training phase, all other parameters remained consistent. The loss curves pertaining to the proposed model under the varied turbulence intensities are delineated in Figure 8. Specifically, the loss function curves corresponding to weak, moderate, and strong turbulence intensities are represented by the blue, red, and green curves, respectively. Notably, the red box highlights the magnified loss function curves post 1200 iterations. Consistent with the simulation results, the loss function demonstrates a downward trend with increasing iterations under experimental conditions.

**Figure 9:**
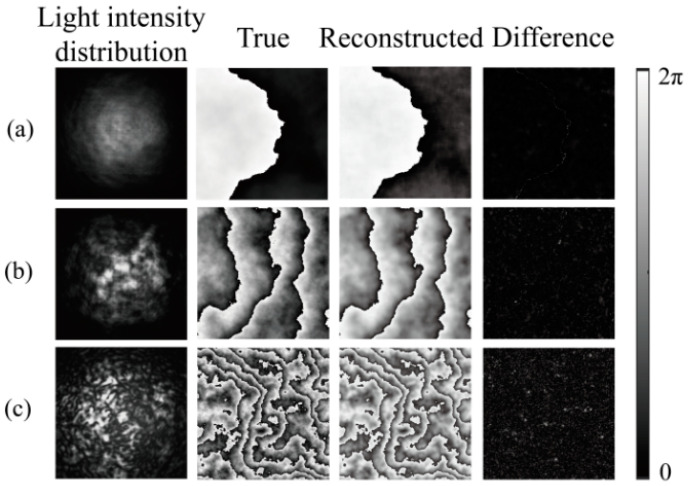
The test results of the proposed model under three different turbulence intensities. (**a**) *C_n_*^2^ = 5 × 10^−17^ m^−2/3^; (**b**) *C_n_*^2^ = 5 × 10^−15^ m^−2/3^; (**c**) *C_n_*^2^ = 1 × 10^−13^ m^−2/3^.

The training loss values are 0.0005, 0.001, and 0.003 under weak, moderate, and strong turbulence intensities, respectively. Moreover, the spikes observed in the loss function curves exhibit greater prominence than simulated counterparts, a phenomenon attributed to the introduction of noise by external environmental factors and experimental equipment during testing. The outcomes of model testing are depicted in Figure 9, where panels (a)–(c) display the test results under weak, moderate, and strong turbulence intensities. The corresponding test loss values for these conditions are 0.00052, 0.0013, and 0.0035, respectively. Each column in Figure 9 illustrates distorted light intensity images under varying turbulence intensities in the first column; real phase screen images in the second column; reconstructed phase screen images by the proposed network model in the third column; and the discrepancy between the second and third columns in the fourth column. These discrepancy images unveil subtle phase fluctuations, indicative of the network model’s challenge in effectively capturing features within regions exhibiting significant phase variations and its susceptibility to noise inherent in the experimental setup. The experimental findings demonstrate the network model’s capability to reconstruct phase screen images from distorted light intensity images across diverse turbulence intensities, underscoring its robust generalization prowess.

## 6. Conclusions

This paper presents a method that uses the U-Net model to reconstruct the atmospheric turbulence phase, and further verified its effectiveness through experiments. Compared to previous turbulence reconstruction schemes based on deep learning, the proposed scheme has decreased the loss function by more than 20 times and reduced time consumption by more than half. The detailed experiment results indicated that the proposed method could effectively extract turbulence phases of different intensities. Moreover, the reconstructive performance of the model remained stable and did not fluctuate with changes in the turbulence intensity, indicating the model’s strong generalization capability. The proposed method allows for the accurate and rapid reconstruction of the wavefront phase under varying turbulence intensities with strong generalization ability and robustness, thereby benefiting the application of free-space coherent optical communication.

## Figures and Tables

**Table 1:** Comparison of reconstruction times of three methods.

Network	Dataset	Reconstruction Time/s	Average Time/s
CNN model [24]	*C_n_*^2^ = 5 × 10^−17^ m^−2/3^ *C_n_*^2^ = 5 × 10^−15^ m^−2/3^ *C_n_*^2^ = 1 × 10^−13^ m^−2/3^	0.42 0.27 0.25	0.31
AlexNet model [25]	*C_n_*^2^ = 5 × 10^−17^ m^−2/3^ *C_n_*^2^ = 5 × 10^−15^ m^−2/3^ *C_n_*^2^ = 1 × 10^−13^ m^−2/3^	0.38 0.26 0.24	0.29
The proposed model	*C_n_*^2^ = 5 × 10^−17^ m^−2/3^ *C_n_*^2^ = 5 × 10^−15^ m^−2/3^ *C_n_*^2^ = 1 × 10^−13^ m^−2/3^	0.18 0.13 0.12	0.14

## Data Availability

The data presented in this study are available on request from the corresponding author.
